# Polymorphisms in *FCN* genes and their influence on systemic lupus erythematosus susceptibility: a report from Western India

**DOI:** 10.1093/immhor/vlaf064

**Published:** 2025-11-24

**Authors:** Kirti Rai, Ridi Khatri, Amrutha Jose, Milind Nadkar, Anjali Rajadhyaksha, Harshada Konkar, Trisha Samant, Pooja Jaiswal, Kunal Dabholkar, Swapnal Pawaskar, Aman Malik, Altaf Parande, Gauthami Bitla, Prashant Tapase, Vijay Padwal, Manisha Madkaikar, Vandana D Pradhan

**Affiliations:** Department of Clinical and Experimental Immunology, National Institute of Immunohaematology, Indian Council of Medical Research, Mumbai, Maharashtra, India; Department of Clinical and Experimental Immunology, National Institute of Immunohaematology, Indian Council of Medical Research, Mumbai, Maharashtra, India; National Institute of Immunohaematology, Indian Council of Medical Research, Mumbai, Maharashtra, India; Department of Medicine, G.S. Medical College and King Edward Memorial Hospital, Mumbai, Maharashtra, India; Department of Medicine, G.S. Medical College and King Edward Memorial Hospital, Mumbai, Maharashtra, India; Department of Clinical and Experimental Immunology, National Institute of Immunohaematology, Indian Council of Medical Research, Mumbai, Maharashtra, India; Department of Clinical and Experimental Immunology, National Institute of Immunohaematology, Indian Council of Medical Research, Mumbai, Maharashtra, India; Department of Clinical and Experimental Immunology, National Institute of Immunohaematology, Indian Council of Medical Research, Mumbai, Maharashtra, India; Department of Clinical and Experimental Immunology, National Institute of Immunohaematology, Indian Council of Medical Research, Mumbai, Maharashtra, India; Department of Clinical and Experimental Immunology, National Institute of Immunohaematology, Indian Council of Medical Research, Mumbai, Maharashtra, India; Department of Clinical and Experimental Immunology, National Institute of Immunohaematology, Indian Council of Medical Research, Mumbai, Maharashtra, India; Department of Clinical and Experimental Immunology, National Institute of Immunohaematology, Indian Council of Medical Research, Mumbai, Maharashtra, India; Department of Clinical and Experimental Immunology, National Institute of Immunohaematology, Indian Council of Medical Research, Mumbai, Maharashtra, India; Information Technology, National Institute of Immunohaematology, Indian Council of Medical Research, Mumbai, Maharashtra, India; Library and Information Technology, National Institute of Immunohaematology, Indian Council of Medical Research, Mumbai, Maharashtra, India; Department of Pediatric Immunology and Leukocyte Biology, National Institute of Immunohaematology, Indian Council of Medical Research, Mumbai, Maharashtra, India; Department of Clinical and Experimental Immunology, National Institute of Immunohaematology, Indian Council of Medical Research, Mumbai, Maharashtra, India

**Keywords:** ficolin gene polymorphisms, ficolin levels, lectin pathway, lupus nephritis, systemic lupus erythematosus

## Abstract

Ficolins, encoded by *FCN* genes, are key pattern recognition molecules of the lectin complement pathway involved in immune complex clearance, a process often impaired in systemic lupus erythematosus (SLE). Genetic polymorphisms in *FCN* genes may influence disease susceptibility. However, their functional significance in SLE remains unclear. The present study aimed to investigate the association of selected *FCN* gene single-nucleotide polymorphisms (SNPs) with SLE, lupus nephritis (LN), and serum ficolin levels in a Western Indian cohort. Seven SNPs in *FCN1* (rs2989727, rs1071583), *FCN2* (rs7851696, rs17549193, rs7865453, rs17514136), and *FCN3* (rs3813800) were genotyped in 200 SLE patients and 200 healthy controls using polymerase chain reaction (PCR) sequence-specific primer and PCR restriction fragment length polymorphism. Serum ficolin-1, -2, and -3 levels were measured using ELISA. Statistical analysis included χ^2^ test, Kruskal–Wallis test, and logistic regression to assess associations and calculate odds ratios with 95% confidence intervals. The analysis identified significant associations of *FCN2* rs7851696, rs7865453, and rs17514136, as well as *FCN3* rs3813800, with SLE susceptibility. Among LN patients, *FCN1* rs2989727 and rs1071583, *FCN2* rs17514136, and *FCN3* rs3813800 showed significant associations. *FCN3* rs3813800 was significantly associated with ficolin-3 levels, while *FCN2* rs7865453 was associated with complement component 1q–circulation immune complex levels. These findings provide novel insight into associations of *FCN* gene polymorphisms with SLE and LN susceptibility, with genotype–phenotype correlations suggesting their biological relevance. Future longitudinal and mechanistic studies are warranted to validate these associations and explore their therapeutic potential.

## Introduction

Systemic lupus erythematosus (SLE) is a multifactorial autoimmune condition marked by immune system dysregulation and generation of autoantibodies against self-antigens. While the exact cause is still not fully understood, a combination of genetic predisposition, environmental exposures, and immune-related mechanisms plays a significant role in the onset and progression of the disease.^1^ Among the key immune pathways involved in SLE, the complement activation plays a central role. There are 3 types of complement activation pathways: classical, alternative, and lectin. Of these, the lectin pathway is regarded as the most intricate and is initiated by pattern recognition molecules (PRMs) such as ficolins.^2^ These molecules bind to pathogens or altered self-structures and associate with mannose-binding lectin-associated serine proteases to initiate the complement cascade.^3^ This pathway is crucial for immune homeostasis by facilitating the removal of apoptotic cells and immune complexes,^3–7^ processes often defective in SLE.

Three distinct types of ficolins have been characterized in humans: ficolin-1 (M-ficolin), predominantly expressed by monocytes and macrophages; ficolin-2 (L-ficolin), primarily produced in the liver and circulating in the serum; and ficolin-3 (H-ficolin or Hakata antigen), synthesized mainly in the liver and lung and secreted into serum and bile.^7–9^ These molecules influence the immune response by recognizing apoptotic cells and pathogens, facilitating their clearance.^5^ This function is critical in preventing the accumulation of immune complexes and apoptotic debris, which can exacerbate autoimmune conditions like SLE. Ficolin-1, encoded by *FCN1*, is primarily expressed in monocytes and neutrophils and circulates at lower concentrations.^2^ Ficolin-2, encoded by *FCN2*, is the second most prevalent serum ficolin, whereas ficolin-3, derived from the *FCN3* gene, is the most abundant in circulation and serves as a key initiator of lectin pathway activation.^2^ Alterations in the concentrations of lectin pathway proteins, including ficolins, correlate with disease activity and specific clinical manifestations in SLE.^10^^,^^11^ The *FCN1* and *FCN2* genes are mapped to the chromosomal regions 9q34 and 9q34.3, respectively, while *FCN3* is situated at 1p36.11.^3–5^^,^^8^^,^^12^ These genes harbor several polymorphisms that may influence susceptibility to SLE or serum ficolin levels.^8^^,^^9^^,^^13–15^ Despite this, their functional significance and clinical implications are poorly understood.

The present study aimed to investigate the association between polymorphisms in the *FCN1*, *FCN2*, and *FCN3* genes and the susceptibility to SLE and their clinical manifestations, particularly lupus nephritis (LN), in patients from Western India. Additionally, the study assessed the association between these gene polymorphisms and their corresponding serum ficolin levels to explore potential genotype–phenotype correlations. Linkage disequilibrium (LD) patterns among the *FCN* single-nucleotide polymorphisms (SNPs) were also analyzed to understand the extent of genetic linkage within these loci.

## Materials and methods

### Study participants

This cross-sectional study included patients with clinically diagnosed SLE (*n* = 200), enrolled between 2021 and 2023 at the Indian Council of Medical Research–National Institute of Immunohaematology (ICMR-NIIH), Mumbai, India, all of whom fulfilled the American College of Rheumatology classification criteria for SLE.^16^ The study received ethical approval from the Institutional Ethics Committee (ICMR-NIIH/IEC/07/2020), and all participants provided written informed consent. The study adhered to the ethical principles of the Declaration of Helsinki. Detailed demographic and clinical data were collected using standardized case record forms. The Safety of Estrogens in Lupus Erythematosus National Assessment–Systemic Lupus Erythematosus Disease Activity Index (SELENA-SLEDAI) scoring system was used to evaluate disease activity.^17^ SLE patients with significant hyperlipidemia, diabetes mellitus, or hypertension and those who were pregnant, postmenopausal, or active smokers were excluded. For comparative analysis, the present study also enrolled unrelated age- and sex-matched healthy controls (HCs) (*n* = 200) with no history of autoimmune disorders. EDTA and plain vials were used for blood collection. Serum was separated and stored at −80 °C until tested.

### Serological analysis

Indirect immunofluorescence technique using HEp-2 cells and *Crithidia lucillae* was used to screen for antinuclear antibody (ANA) and anti-dsDNA autoantibodies, respectively (EUROIMMUN, Germany). The ANA profile was detected by the LINE Blot assay (EUROIMMUN). C3 and C4 complement levels were determined using the MISPA-i3 Nephelometer (Agappe, Kerala, India). The reference cut-off values used for interpretation were 80–180 mg% for C3 and 10–40 mg% for C4. Complement component 1q–circulation immune complex (C1q-CIC) levels were estimated using the C1q-CIC enzyme-linked immunosorbent assay (ELISA) kit (EIA-3169, DRG International, USA), with reference cut-off values of ≤16 μgEq/mL (negative sample) and >16 μgEq/mL (positive sample). The serum levels of ficolin-1, ficolin-2, and ficolin-3 were quantified using ELISA kits, procured from Hycult Biotech (Netherlands), with catalog numbers HK357, HK336, and HK340, respectively. The reference cut-off values used for interpretation were as follows: ficolin-1: 0 to 788.00 ng/mL (normal) and >788.00 ng/mL (high); ficolin-2: 0 to 1,929.63 ng/mL (normal) and >1,929.63 (high); and ficolin-3: 0 to 33,852.12 ng/mL (normal) and >33,852.12 ng/mL (high). These cut-offs for ficolin levels were established by receiver operating characteristic (ROC) curve analysis using data from HCs (*n* = 90).

### *FCN* genotyping

The QIAGEN-FlexiGene Extraction (Qiagen, Germany) kit was used for extraction of genomic DNA from 300 μL whole blood. The extracted DNA was stored at −20°C until tested. Seven SNPs within the *FCN1*, *FCN2*, and *FCN3* genes were selected based on their functional relevance and prior associations with disease susceptibility.^18^^,^^19^ Genotyping of all SLE patients and HCs was performed to detect 2 polymorphisms in the *FCN1* gene at position −1981 G > A (rs2989727), and +7919 A > G (rs1071583); 4 polymorphisms in the *FCN2* gene at position +6424 G > T (rs7851696), +6359 C > T (rs17549193), −64 A > C (rs7865453), and −4 A > G (rs17514136); and one polymorphism in the *FCN3* gene at position +3836 C > G (rs3813800). Genotyping of rs2989727, rs7851696, and rs17549193 was conducted using the PCR sequence-specific primer technique as described by Szala et al.^20^ The remaining polymorphisms were examined using the PCR restriction fragment length polymorphism using restriction enzymes StyI, Hpy188I, MboII, and AciI, respectively. All of the PCR assays began with a denaturation step at 95 °C for 5 minutes, proceeded through amplification cycles, and terminated with an extension step of 5 to 7 minutes at 72 °C. The reaction was performed in a final volume of 25 μL using the primers listed in Table 1.

**Table 1.** Primer sequences used for the study experiments.

Gene	Primer sequence
*FCN1* −1981G > A (rs2989727)	FCN1 Prom_−1981AF: 5′-CCCATGAGCCTGGTTATCA-3′ FCN1 Prom_−1981GR: 5′-CCCATGAGCCTGGTTATCG-3′ FCN1_Common R: 5′-ACCTCCTCTTCCTTGCAACA-3′
*FCN1* +7919A > G (rs1071583)	F: 5′-TGGAGGTGGAAAATCCTCGC-3′ R: 5′-AAGTGAGGTTGGCTCTGCTC-3′
*FCN2* +6424G > T (rs7851696)	P6424_FG: 5′-GATCTTAACACCGGAAATGGTG-3′ P6424_FT: 5′-GATCTTAACACCGGAAATAGTT-3′ P6424_Ctrl: 5′-ACGATGCTCACATTTCCTCC-3′ P6424_Rev: 5′-TTACAAACCGTAGGGCCAAG-3′
*FCN2* +6359C > T (rs17549193)	P6359_F1: 5′-TTGCACTTCTTGGATTGTGC-3′ P6359_F2: 5′-CCTGCACAGGAGATTCCCGGAT-3′ P6359_R1: 5′-GGACTGGTTGTTGTTGAACG-3′ P6359_R2: 5′-TGGCAGTTTTTGTACCACCA-3′
*FCN2* −64A > C (rs7865453)	F: 5′-CTACAGGTGGGATGTCTGGC-3′ R: 5′-GAAGCCACCAATCACGAAGC-3′
*FCN2* −4A > G (rs17514136)	F: 5′-CTACAGGTGGGATGTCTGGC-3′ R: 5′-GAAGCCACCAATCACGAAGC-3′
*FCN3* +3836C > G (rs3813800)	F: 5′-AGTGGGCGAAAGTACGGTTAC-3′ R: 5′-TTTATCAACAGGCCCCAATCC-3′

### Statistical analysis

Statistical analyses were performed using IBM SPSS Statistics version 27 software. Continuous variables are presented as medians with interquartile ranges (IQRs), and categorical variables are summarized as frequencies and percentages. Hardy–Weinberg equilibrium in both patients and HCs was assessed using Pearson χ^2^ test with the Social Science Statistics tool (https://www.socscistatistics.com). Comparisons of genotype distributions and allele frequencies between groups were conducted using the χ^2^ test. Odds ratios (ORs) with corresponding 95% confidence intervals (CIs) were estimated using logistic regression models. Kruskal–Wallis test, followed by Dunn multiple comparisons test, was applied to compare ficolin levels across genotypes. ROC curve analysis and the Youden index were used to determine the cut-offs for ficolin levels. The LD between ficolin SNPs was analyzed using Haploview version 4.1 software (https://www.broadinstitute.org/haploview/haploview). A *P* value <0.05 was considered statistically significant.

## Results

### Patient demographics and clinical characteristics

The present study enrolled patients with clinically diagnosed SLE (*n* = 200), with a median age of 31 years (IQR, 23 to 41 years) at enrollment. The median age of disease onset was 28 years (IQR, 21 to 38 years). The cohort consisted predominantly of females (89.5%), while males accounted for 10.5%, resulting in a female-to-male ratio of approximately 8.5:1. The median SELENA-SLEDAI score was 7 (IQR, 4 to 11) at the time of patient enrollment. Among clinical manifestations, mucocutaneous involvement was the most common (62.0%), followed by constitutional symptoms (41.5%), hematological abnormalities (35.0%), and LN (34.0%). Musculoskeletal (19.0%), neuropsychiatric (4.0%), and serosal involvement (3.5%) were less frequently reported. Laboratory findings revealed that 95% of patients were ANA positive, while 80% were anti-dsDNA autoantibody positive. Low C3 levels were noted in 53.5%, and low C4 levels were noted in 56.0% of patients. Among the SLE patients, 112 (56.0%) were untreated at the time of evaluation, while 88 (44.0%) had received treatment. Among those treated, hydroxychloroquine was the most commonly prescribed medication, administered to 69 patients (78.4%), followed by prednisone in 65 patients (73.9%). Cyclophosphamide was given to 32 patients (36.4%), and azathioprine to 9 patients (10.2%). Table 2 summarizes the demographic and clinical characteristics of the SLE patients included in this study. The median age of HCs (*n* = 200) was 29 years (IQR, 23 to 38 years). The HC group included 165 females (82.5%) and 35 males (17.5%).

**Table 2.** Demographic, clinical, and laboratory characteristics of SLE patients (*n* = 200).

Characteristic	Value	
Demographic characteristics (*n* = 200)		
Age at enrollment, years	31 (23, 41)	
Age of onset, years	28 (21, 38)	
Female	179 (89.5)	
Male	21 (10.5)	
Duration of treatment, months	6 (1, 36)	
SLEDAI score	7 (4, 11)	
Clinical manifestations		
Mucocutaneous	124 (62.0)	
Constitutional	83 (41.5)	
Hematological	70 (35.0)	
Lupus nephritis	68 (34.0)	
Musculoskeletal	38 (19.0)	
Neuropsychiatric	8 (4.0)	
Serosal (pleuritis and/or pericarditis)	7 (3.5)	
Laboratory parameters		
ANA positivity	190 (95.0)	
Anti-dsDNA positivity	160 (80.0)	
Low C3	107 (53.5)	
Low C4	112 (56.0)	
Low C3 and C4	81 (40.5)	
Ficolin-1 (ng/mL)	477.18 (213.00, 916.73)	
Ficolin-2 (ng/mL)	3,065.87 (1,702.50, 5,398.64)	
Ficolin-3 (ng/mL)	3,6318.45 (9,485.50, 72,938.01)	
C1q-CIC (μgEq/mL)	13.34 (5.43, 25.44)	
Treatment		
Treatment status	Untreated SLE	Treated SLE
	112 (56.0)	88 (44.0)
Hydroxychloroquine	…	69 (78.4)
Prednisone	…	65 (73.9)
Cyclophosphamide	…	32 (36.4)
Azathioprine	…	9 (10.2)

Continuous variables expressed as median (IQR) and categorial data as frequency (%).

### Comparison of *FCN* gene polymorphisms among SLE patients and HCs

The comparison of *FCN1*, *FCN2*, and *FCN3* gene polymorphisms among SLE patients and HCs is shown in Table 3. The genotype distributions for *FCN1*, *FCN2* rs7851696, and *FCN3* SNPs were consistent with Hardy–Weinberg equilibrium, whereas deviations were observed for *FCN2* rs17549193, rs7865453, and rs17514136 genotypes. For *FCN1*, genotype frequencies of rs2989727 and rs1071583 did not differ significantly between SLE patients and HCs. However, the frequency of the minor G allele of *FCN1* rs1071583 was significantly less among SLE patients (74.0%) compared with HCs (80.5%) (OR, 0.69 [95% CI, 0.49 to 0.96; *P* = 0.028]). For *FCN2* rs17549193, the difference between genotype and allele frequencies among SLE patients and HCs was not statistically significant. For *FCN2* rs7851696, the TT genotype frequency was higher among SLE patients (10.0%) compared with HCs (3.0%) (OR, 3.59 [95% CI, 1.41 to 9.15]; *P* *=* 0.005), along with a higher frequency of the minor T allele among SLE patients (27.0%) compared with HCs (21.0%) (OR, 1.39 [95% CI, 1.00 to 1.93]; *P* *=* 0.047). In contrast, the CC genotype of *FCN2* rs7865453 was significantly less frequent among SLE patients (16.0%) compared with HCs (27.0%) (OR, 0.51 [95% CI, 0.32 to 0.84]; *P* *=* 0.007), and a lesser frequency of the minor C allele was noted among SLE patients (23.2%) compared with HCs (33.0%) (OR, 0.62 [95% CI, 0.45 to 0.84]; *P* *=* 0.002). For *FCN2* rs17514136, the heterozygous AG genotype was more common among SLE patients (29.5%) compared with HCs (7.0%) (OR, 5.56 [95% CI, 2.98 to 10.36]; *P* *<* 0.001), whereas the AA genotype was less frequent among SLE patients (55.5%) than HCs (84.0%) (OR, 0.24 [95% CI, 0.15 to 0.38]; *P* *<* 0.001). Additionally, the minor G allele frequency was significantly higher among SLE patients (29.7%) compared with HCs (12.5%) (OR, 2.96 [95% CI, 2.06 to 4.27]; *P* *<* 0.001). For *FCN3*, the heterozygous CG genotype of rs3813800 was more frequent among SLE patients (36.5%) than HCs (27.0%) (OR, 1.55 [95% CI, 1.02 to 2.38]; *P* *=* 0.041), while the CC genotype was significantly less frequent among SLE patients (11.5% vs. 26.0%) (OR, 0.37 [95% CI, 0.22 to 0.63]; *P* *<* 0.001). Additionally, the minor G allele was more common among SLE patients (70.2%) as compared to HCs (60.5%) (OR, 1.54 [95% CI, 1.15 to 2.07]; *P* *=* 0.004).

**Table 3.** Comparison of *FCN* gene polymorphism genotypes and allele frequencies in SLE patients (*n* = 200) and HCs (*n* = 200).

SNPs	SLE (*n* = 200)	HCs (*n* = 200)	OR (95% CI)	*P* value
*FCN1* rs2989727 (−1981G > A)				
GG	28 (14.0)	32 (16.0)	0.85 (0.49, 1.48)	0.575
GA	105 (52.5)	102 (51.0)	1.06 (0.72, 1.57)	0.764
AA	67 (33.5)	66 (33.0)	1.02 (0.67, 1.55)	0.915
Alleles				
G allele	161 (40.3)	166 (41.5)	0.95 (0.72, 1.26)	
A allele	239 (59.7)	234 (58.5)	1.05 (0.79, 1.40)	0.719
*FCN1* rs1071583 (+7919A > G)				
AA	15 (7.5)	8 (4.0)	1.95 (0.81, 4.70)	0.133
AG	74 (37.0)	62 (31.0)	1.31 (0.86, 1.98)	0.205
GG	111 (55.5)	130 (65.0)	0.67 (0.45, 1.01)	0.052
Alleles				
A allele	104 (26.0)	78 (19.5)	1.45 (1.04, 2.02)	
G allele	296 (74.0)	322 (80.5)	0.69 (0.49, 0.96)	**0.028**
*FCN2* rs7851696 (+6424G > T)				
GG	112 (56.0)	122 (61.0)	0.81 (0.55, 1.21)	0.310
GT	68 (34.0)	72 (36.0)	0.92 (0.61, 1.38)	0.675
TT	20 (10.0)	6 (3.0)	3.59 (1.41, 9.15)	**0.005**
Alleles				
G allele	292 (73.0)	316 (79.0)	0.72 (0.52, 1.00)	
T allele	108 (27.0)	84 (21.0)	1.39 (1.00, 1.93)	**0.047**
*FCN2* rs17549193 (+6359C > T)				
CC	67 (33.5)	56 (28.0)	1.30 (0.85, 1.98)	0.233
CT	61 (30.5)	64 (32.0)	0.93 (0.61, 1.42)	0.746
TT	72 (36.0)	80 (40.0)	0.84 (0.56, 1.26)	0.410
Alleles				
C allele	195 (48.8)	176 (44.0)	1.21 (0.92, 1.60)	
T allele	205 (51.2)	224 (56.0)	0.83 (0.63, 1.09)	0.178
*FCN2* rs7865453 (−64A > C)				
AA	139 (69.5)	122 (61.0)	1.46 (0.96, 2.20)	0.074
AC	29 (14.5)	24 (12.0)	1.24 (0.70, 2.22)	0.461
CC	32 (16.0)	54 (27.0)	0.51 (0.32, 0.84)	**0.007**
Alleles				
A allele	307 (76.8)	268 (67.0)	1.63 (1.19, 2.22)	
C allele	93 (23.2)	132 (33.0)	0.62 (0.45, 0.84)	**0.002**
*FCN2* rs17514136 (−4A > G)				
AA	111 (55.5)	168 (84.0)	0.24 (0.15, 0.38)	**<0.001**
AG	59 (29.5)	14 (7.0)	5.56 (2.98, 10.36)	**<0.001**
GG	30 (15.0)	18 (9.0)	1.78 (0.96, 3.32)	0.065
Alleles				
A allele	281 (70.3)	350 (87.5)	0.34 (0.23, 0.49)	
G allele	119 (29.7)	50 (12.5)	2.96 (2.06, 4.27)	**<0.001**
*FCN3* rs3813800 (+3836C > G)				
CC	23 (11.5)	52 (26.0)	0.37 (0.22, 0.63)	**<0.001**
CG	73 (36.5)	54 (27.0)	1.55 (1.02, 2.38)	**0.041**
GG	104 (52.0)	94 (47.0)	1.22 (0.83, 1.81)	0.317
Alleles				
C allele	119 (29.8)	158 (39.5)	0.65 (0.48, 0.87)	
G allele	281 (70.2)	242 (60.5)	1.54 (1.15, 2.07)	**0.004**

Data are presented as frequency (%). Genotype and allele frequencies were compared between the groups using the χ^2^ test. ORs with 95% CIs were estimated using logistic regression. A *P* value <0.05 was considered statistically significant. Values in bold indicate statistically significant results.

### Association of *FCN* gene polymorphisms among SLE patients with LN

The association of *FCN1*, *FCN2*, and *FCN3* gene polymorphisms in SLE patients with or without LN is shown in Table 4. For *FCN1*, the GG genotype of rs2989727 was significantly less frequent among LN patients (3.0%) compared with those without LN (19.7%) (OR, 0.12 [95% CI, 0.03 to 0.54]; *P* *=* 0.001), whereas the AA genotype was notably more common among LN patients (48.5%) than non-LN patients (25.8%) (OR, 2.72 [95% CI, 1.47 to 5.03]; *P* *=* 0.001). Additionally, the minor A allele frequency was significantly higher among LN patients (72.8% vs. 53.0%; OR, 2.37 [95% CI, 1.51 to 3.71]; *P* *<* 0.001). Further, the frequency of the AA genotype of *FCN1* rs1071583 was significantly higher among LN patients (13.2%) than in those without LN (4.5%) (OR, 3.20 [95% CI, 1.09 to 9.42]; *P* *=* 0.027). The minor G allele frequency was lower among LN patients (66.9%) as compared to non-LN patients (77.6%) (OR, 0.58 [95% CI, 0.37 to 0.92]; *P* *=* 0.020). For *FCN2*, no statistically significant differences were observed in genotype or allele frequencies for rs7851696, rs17549193, or rs7865453 between LN and non-LN patients. However, for *FCN2* rs17514136, the frequency of the heterozygous AG genotype was significantly higher among LN patients (39.7%) compared with non-LN patients (24.2%) (OR, 2.06 [95% CI, 1.10–3.86]; *P* *=* 0.023), while the AA genotype was less frequent among LN patients (44.1% vs. 61.4%; OR, 0.50 [95% CI, 0.28 to 0.90]; *P* *=* 0.020). A higher frequency of the minor G allele was observed among LN patients (36.0%) compared with non-LN patients (26.5%) (OR, 1.56 [95% CI, 1.00 to 2.43]; *P* *=* 0.049). For *FCN3*, the frequency of the GG genotype of rs3813800 was significantly less among LN patients (39.7%) compared with non-LN patients (58.3%) (OR, 0.47 [95% CI, 0.26 to 0.85]; *P* *=* 0.012), in addition to a low frequency of the minor G allele among LN patients (61.8%) compared with non-LN patients (74.6%) (OR, 0.55 [95% CI, 0.35 to 0.86]; *P* *=* 0.008).

**Table 4.** Association of *FCN* genotypes and allele frequencies with lupus nephritis.

SNPs	LN (n = 68)	Non-LN (n = 132)	OR (95% CI)	*P* value
*FCN1* rs2989727 (−1981G > A)				
GG	2 (3.0)	26 (19.7)	0.12 (0.03, 0.54)	**0.001**
GA	33 (48.5)	72 (54.5)	0.79 (0.44, 1.41)	0.420
AA	33 (48.5)	34 (25.8)	2.72 (1.47, 5.03)	**0.001**
Alleles				
G allele	37 (27.2)	124 (47.0)	0.42 (0.27, 0.66)	
A allele	99 (72.8)	140 (53.0)	2.37 (1.51, 3.71)	**<0.001**
*FCN1* rs1071583 (+7919A > G)				
AA	9 (13.2)	6 (4.5)	3.20 (1.09, 9.42)	**0.027**
AG	27 (39.7)	47 (35.6)	1.19 (0.65, 2.18)	0.569
GG	32 (47.1)	79 (59.9)	0.60 (0.33, 1.08)	0.085
Alleles				
A allele	45 (33.1)	59 (22.4)	1.72 (1.08, 2.72)	
G allele	91 (66.9)	205 (77.6)	0.58 (0.37, 0.92)	**0.020**
*FCN2* rs7851696 (+6424G > T)				
GG	38 (55.9)	74 (56.1)	0.99 (0.55, 1.79)	0.981
GT	23 (33.8)	45 (34.1)	0.99 (0.53, 1.83)	0.970
TT	7 (10.3)	13 (9.8)	1.05 (0.40, 2.77)	0.921
Alleles				
G allele	99 (72.8)	193 (73.1)	0.98 (0.62, 1.57)	
T allele	37 (27.2)	71 (26.9)	1.02 (0.64, 1.62)	0.947
*FCN2* rs17549193 (+6359C > T)				
CC	20 (29.4)	47 (35.6)	0.75 (0.40, 1.42)	0.379
CT	20 (29.4)	41 (31.1)	0.92 (0.49, 1.75)	0.810
TT	28 (41.2)	44 (33.3)	1.40 (0.77, 2.56)	0.274
Alleles				
C allele	60 (44.1)	135 (51.1)	0.75 (0.50, 1.14)	
T allele	76 (55.9)	129 (48.9)	1.33 (0.87, 2.01)	0.183
*FCN2* rs7865453 (−64A > C)				
AA	50 (73.5)	89 (67.4)	1.34 (0.70, 2.57)	0.374
AC	6 (8.8)	23 (15.2)	0.46 (0.18, 1.19)	0.102
CC	12 (17.7)	20 (17.4)	1.20 (0.55, 2.63)	0.648
Alleles				
A allele	106 (77.9)	201 (76.1)	1.11 (0.68, 1.82)	
C allele	30 (22.1)	63 (23.9)	0.90 (0.55, 1.48)	0.686
*FCN2* rs17514136 (−4A > G)				
AA	30 (44.1)	81 (61.4)	0.50 (0.28, 0.90)	**0.020**
AG	27 (39.7)	32 (24.2)	2.06 (1.10, 3.86)	**0.023**
GG	11 (16.2)	19 (14.4)	1.15 (0.51, 2.57)	0.738
Alleles				
A allele	87 (64.0)	194 (73.5)	0.64 (0.41, 1.00)	
G allele	49 (36.0)	70 (26.5)	1.56 (1.00, 2.43)	**0.049**
*FCN3* rs3813800 (+3836C > G)				
CC	11 (16.2)	12 (9.1)	1.93 (0.80, 4.64)	0.137
CG	30 (44.1)	43 (32.6)	1.63 (0.90, 2.98)	0.108
GG	27 (39.7)	77 (58.3)	0.47 (0.26, 0.85)	**0.012**
Alleles				
C allele	52 (38.2)	67 (25.4)	1.82 (1.17, 2.84)	
G allele	84 (61.8)	197 (74.6)	0.55 (0.35, 0.86)	**0.008**

Data are expressed as frequency (%). Genotype and allele frequencies were compared between the groups using the χ^2^ test. ORs with 95% CIs were estimated using logistic regression. A *P* value <0.05 was considered statistically significant. Values in bold indicate statistically significant results.

### Association of serum ficolin levels with *FCN* genotypes in SLE patients

**Figure 1.**
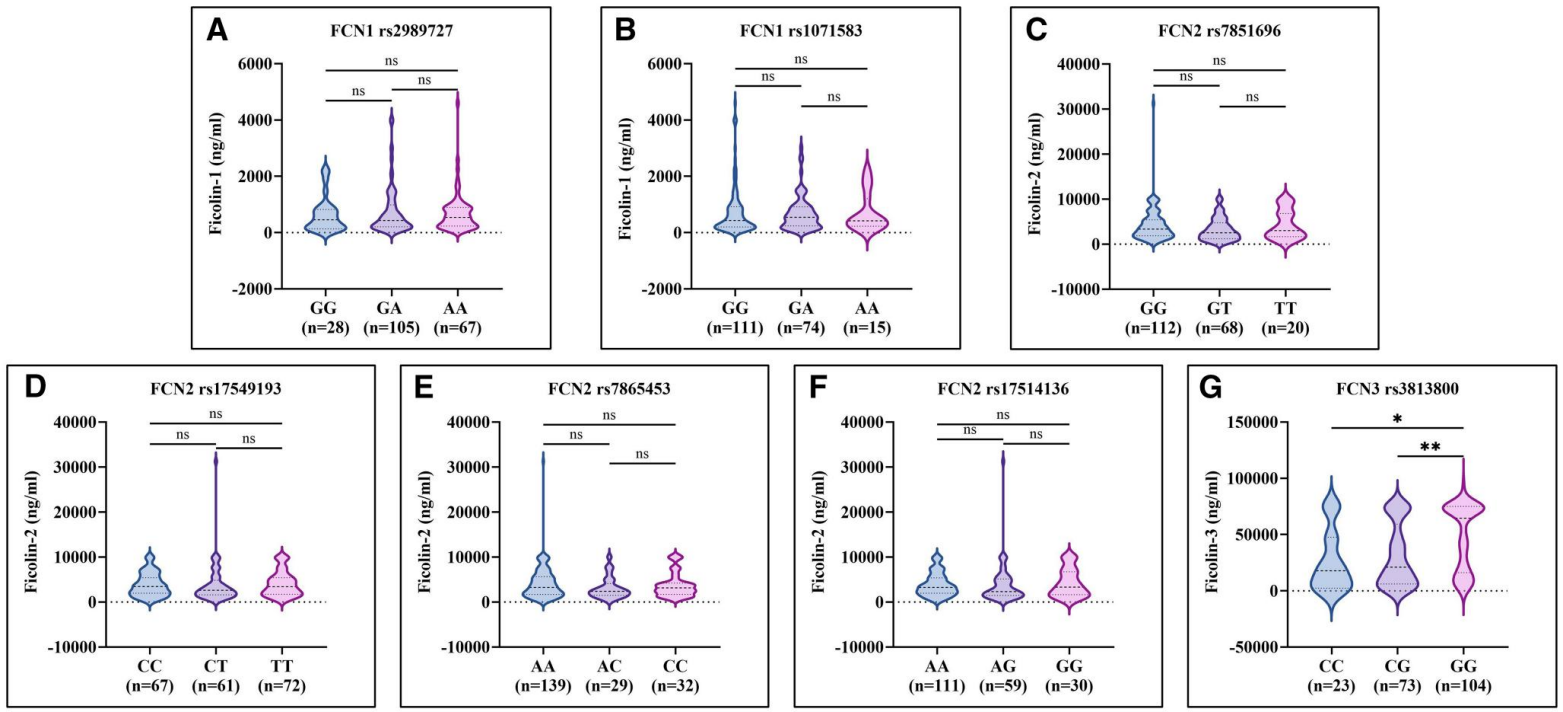
(A–G) Comparison of serum ficolin levels with *FCN* genotypes among SLE patients studied (*n* = 200). Data are presented as violin plots showing distribution, median, and interquartile range. Group comparisons were performed using the Kruskal–Wallis test followed by Dunn multiple comparisons test. Statistical significance is indicated as *P *<* 0.05, **P *<* 0.01; ns, not significant.

The violin plots (Fig. 1A–G) illustrate the distribution of serum ficolin levels (ficolin-1, ficolin-2, and ficolin-3) across different genotypes of *FCN1*, *FCN2*, and *FCN3* polymorphisms in SLE patients. For the *FCN3* rs3813800 (3836C > G) polymorphism, individuals with the GG genotype showed a significant increase in serum ficolin*-*3 levels as compared to those with the CG (*P* *=* 0.001) and CC genotypes (*P* *=* 0.011) (Fig. 1G). For all other *FCN* polymorphisms, the respective ficolin levels were comparable across different genotypes.

### Association of serum C1q-CIC levels with *FCN* genotypes in SLE patients

**Figure 2.**
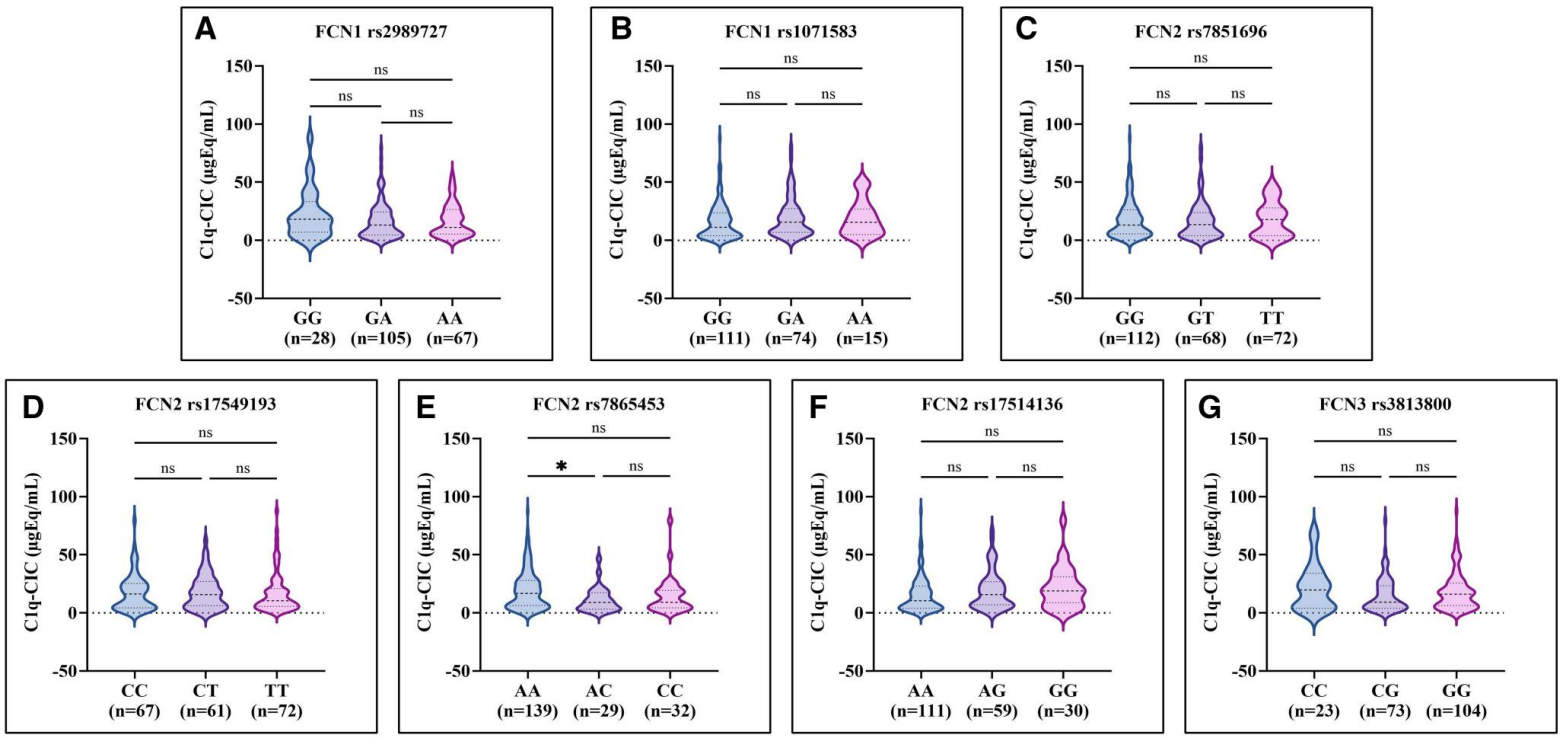
(A–G) Comparison of serum C1q-CIC levels with *FCN* genotypes among SLE patients studied (*n* = 200). Data are presented as violin plots showing distribution, median, and interquartile range. Group comparisons were performed using the Kruskal–Wallis test followed by Dunn multiple comparisons test. Statistical significance is indicated as *P *<* 0.05; ns, not significant.

For the *FCN2* rs7865453 polymorphism, serum C1q-CIC levels were significantly elevated in individuals with the AA genotype compared to those with the AC genotype (*P* *=* 0.010). For all other *FCN* polymorphisms, the respective C1q-CIC levels were comparable across different genotypes (Fig. 2A–G).

### LD Analysis of 6 SNPs across the *FCN* gene cluster in SLE patients and HCs

**Figure 3.**
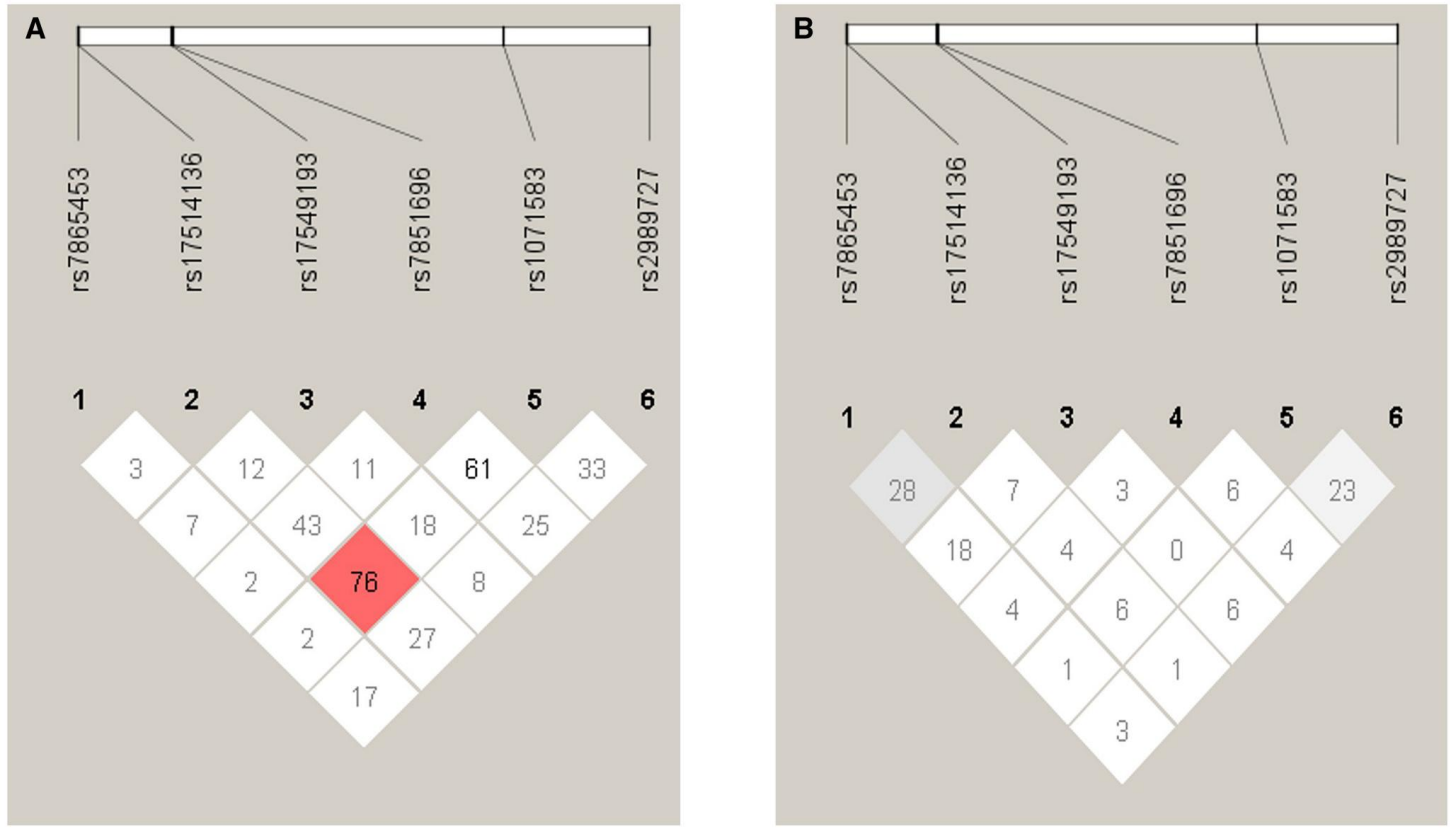
LD plots of (A) HCs and (B) SLE patients for 6 SNPs across *FCN1* and *FCN2* genes indicating the D′ values.

LD analysis of 6 SNPs spanning the *FCN1* and *FCN2* genes was performed in both HCs and SLE patients (Fig. 3A, B). Both D′ and *r*^2^ values were low across most SNP pairs in both groups, indicating limited LD and low allelic correlation. While the D′ between *FCN2* rs17514136 and *FCN1* rs1071583 was 76% in HCs, the corresponding *r*^2^ was low (*r*^2^ = 0.02), suggesting that the alleles are not strongly correlated and therefore not in strong LD.

## Discussion

The *FCN* genes encode ficolins, which are soluble PRMs that activate the complement system via the lectin pathway. These genes are highly polymorphic and are located on distinct chromosomal loci. However, the clinical and functional significance of these genetic polymorphisms remains incompletely understood. In particular, the genotype–phenotype correlations have not been fully characterized for understanding their influence on SLE disease susceptibility. Given this, the present study investigated the association of some SNPs in the *FCN1*, *FCN2*, and *FCN3* genes with susceptibility to SLE, renal manifestations (as seen in LN), and serum ficolin levels. The findings revealed significant genetic variations within the *FCN* gene cluster that were associated with SLE susceptibility and disease heterogeneity. Notably, specific SNPs in *FCN1* (rs2989727 and rs1071583), *FCN2* (rs17514136), and *FCN3* (rs3813800) genes showed strong associations with the presence of LN, indicating a potential link with renal manifestations of SLE. Importantly, the *FCN2* rs7865453 and *FCN3* rs3813800 polymorphisms were associated with differences in serum C1q-CIC levels and ficolin-3 levels, respectively, suggesting possible functional relevance.

The present study investigated 2 *FCN1* SNPs, rs2989727 and rs1071583, and their susceptibility to SLE. For both these SNPs, the genotype frequencies did not differ significantly among SLE patients and HCs. This suggests that *FCN1* SNPs may not be associated with overall susceptibility for SLE in the Indian cohort. Importantly, both of these SNPs showed a significant association with the occurrence of LN. The high occurrence of the AA genotype and the minor A allele of *FCN1* rs2989727, as well as the AA genotype and the major A allele of *FCN1* rs1071583, among LN patients suggests that *FCN1* variants may be associated with renal involvement in SLE and therefore could serve as a potential marker for predicting renal complications in SLE. A previous study by Addobbati et al. conducted in a Brazilian population reported no significant associations between these SNPs and SLE or LN.^8^ This suggests potential population-specific genetic influences on SLE disease susceptibility and organ-specific manifestations.

To further determine the functional relevance of these SNPs, the serum levels of ficolin-1 were compared across different *FCN* genotypes. The present study showed that these specific SNPs may not directly influence ficolin-1 levels in SLE patients. This finding was similar to a previous study by Munthe-Fog et al.^21^ However, the same study reported that other promoter region polymorphisms, namely *FCN1* rs10120023 and *FCN1* rs10117466, were linked to altered ficolin-1 levels.^21^ Some other studies suggested the potential immunogenetic relevance of *FCN1* polymorphisms in other autoimmune conditions. The *FCN1* gene variants, particularly rs2989727 and rs1071583, have been reported to be linked to increased risk of developing rheumatoid arthritis in Brazilian and Belgian populations, whereas no such associations were observed for *FCN2* or *FCN3* variants.^22^ The *FCN1* gene polymorphisms have also been reported in other diseases such as pneumonia^23^ and rheumatic fever and rheumatic heart disease.^24^

For the *FCN2* gene, the distribution of 4 SNPs—namely rs7851696, rs17549193, rs7865453, and rs17514136—is reported in the present study. For *FCN2* rs7851696, the TT genotype and the minor T allele and for *FCN2* rs17514136, the AG genotype and the minor G allele were more prevalent among SLE patients compared with HCs. This distribution suggests the potential association with disease susceptibility. In contrast, for *FCN2* rs7865453, the CC genotype and the minor C allele at a lower frequency were noted in SLE patients. Notably, for *FCN2* rs17514136, the AG genotype and the minor G allele were linked with higher risk of developing LN, indicating a possible association with LN susceptibility. These findings were similar to those of earlier reports, which demonstrated the associations of *FCN2* polymorphisms with SLE susceptibility and clinical manifestations. A study by Elkoumi et al. in patients with pediatric-onset SLE (pSLE) reported that *FCN2* promoter region polymorphisms may influence pSLE susceptibility, where rs3124952 and rs17514136 were significantly associated with increased risk of LN.^14^ Similarly, Addobbati et al. reported that the *FCN2* rs17514136 SNP was linked with severe SLE disease activity, while *FCN2* rs3124954 SNP was associated with LN.^8^ A study in pSLE patients from Egypt reported that the *FCN2* rs3124954 SNP influenced the disease.^25^ These findings suggest that the possible role of *FCN2* gene polymorphisms may be associated with SLE risk and clinical outcomes.

The present study also evaluated the association of these SNPs with ficolin-2 levels, indicating that these SNPs may not be directly associated with variations in ficolin-2 expression. These findings were in contrast to the study by Elkoumi et al., who reported that the *FCN2* rs3124952 and rs17514136 SNPs were associated with altered ficolin-2 levels in pSLE patients.^14^ Another study in Danish Caucasians had reported associations between *FCN2* gene polymorphisms and the levels of ficolin-2.^26^ These findings suggested that age of onset, population-specific factors, and SNP locations may influence the genotype–phenotype relationships. Additionally, the present study identified a significant association between the *FCN2* rs7865453 SNP and elevated C1q-CIC levels in individuals with the AA genotype compared to the AC genotype. This suggests a possible association of this SNP with immune complex clearance in SLE. However, additional research is needed to investigate the mechanistic basis of this association and its clinical implications in SLE disease activity and pathogenesis.

For *FCN3*, the rs3813800 SNP in the present study showed that the heterozygous CG genotype and the minor G allele were significantly more prevalent in SLE patients, indicating a potential association with increased susceptibility to SLE. Furthermore, the low frequency of the GG genotype and the minor G allele in patients with LN in comparison to those without LN suggests its possible protective role in LN.

The *FCN3* rs3813800 polymorphism was associated with differences in circulating ficolin-3 levels. Individuals with the GG genotype exhibited significantly elevated serum ficolin-3 levels as compared to those with CG and CC genotypes. This suggested that this polymorphism may be linked to both genetic predisposition and differences in ficolin-3 levels. However, further studies are warranted to explore its mechanistic role. A recent study by Lindelöf et al. reported that the *FCN3* rs2474288 SNP influenced ficolin-3 levels in Swedish SLE patients.^13^ Another study by Troldborg et al. in a Danish population reported that ficolin-3 deficiency, caused by the *FCN3* +1637delC mutation, was linked to an increased risk of SLE but that heterozygosity did not increase SLE risk.^15^

Haploview analysis revealed overall weak LD across both the SLE patients and the HCs. The absence of strong LD blocks in both the groups suggests a lack of a tightly linked haplotype structure in this genomic region among the 6 SNPs and is indicative of historical recombination events and independent inheritance pattern of the alleles.^27^

In the present study, reductions in complement components C3 and C4 were observed in more than half of the SLE patients and were significantly associated with disease activity. The complement system plays a crucial role in immune complex clearance and maintaining immune homeostasis. Deficiencies and consumption of the complement components C3 and C4 are well-documented features in SLE and are considered both markers and mediators of disease activity.^28^ Hypocomplementemia is reported to be associated with various clinical manifestations including LN. Our findings indicated a significant association between reduced C3 and C4 levels and disease activity, supporting the clinical utility of monitoring these complement components for effective disease management.

To the best of our knowledge, this study is the first to comprehensively assess the associations of *FCN1*, *FCN2*, and *FCN3* gene polymorphisms with SLE susceptibility, renal involvement, and serum ficolin levels in an Indian population. This integrated genetic and serological approach provides novel insight into the potential functional relevance of *FCN* gene variants in SLE. Most of the earlier studies have focused on the classical complement pathway in SLE pathogenesis, but the present study highlights the impact of the lectin pathway, particularly ficolins, in disease susceptibility and renal manifestations. The present study also has some limitations. Being a study in a single ethnic population, it may restrict the population-based variations among different ethnic backgrounds across the country. In the present study, the mRNA expression levels and downstream complement activation were not assessed. This would have provided more comprehensive insight into functional consequences. The environmental or epigenetic factors that could influence ficolin expression were not explored in the present study.

In conclusion, the present study provides a novel insight into the association of *FCN* gene polymorphisms with disease susceptibility, renal involvement, and serum ficolin levels in an Indian cohort. The observed associations of *FCN1* (rs2989727 and rs1071583), *FCN2* (rs17514136), and *FCN3* (rs3813800) SNPs with LN highlight a potential link between the lectin complement pathway in renal manifestations of SLE. Moreover, the associations between *FCN2* rs7865453 genotypes and serum C1q-CIC levels as well as *FCN3* rs3813800 genotypes and serum ficolin-3 levels suggest the biological relevance of these polymorphisms. However, future longitudinal and mechanistic studies are warranted to validate these associations and explore their utility as possible biomarkers or therapeutic targets in SLE.

## Data Availability

The datasets used and/or analyzed during the current study are available from the corresponding author upon request.

## Abbreviations

ANA: antinuclear antibody
C1q-CIC: complement component 1q–circulation immune complex
CI: confidence interval
ELISA: enzyme-linked immunosorbent assay
FCN: ficolin
HCs: healthy controls
IQR: interquartile range
LD: linkage disequilibrium
LN: lupus nephritis
OR: odds ratio
PCR: polymerase chain reaction
PRM: pattern recognition molecule
pSLE: pediatric-onset systemic lupus erythematosus
ROC: receiver operating characteristic
SELENA-SLEDAI: Safety of Estrogens in Lupus Erythematosus National Assessment–Systemic Lupus Erythematosus Disease Activity Index
SLE: systemic lupus erythematosus
SNP: single-nucleotide polymorphism
